# Behavioral and Electrophysiological Evidence for Repellency of *Phytolacca americana* (Pokeweed) Fruit Extract in *Plutella xylostella*

**DOI:** 10.3390/insects17060641

**Published:** 2026-06-17

**Authors:** Yang Liu, Yong-Hao Zhai, Chen-Han Sun, Jia-Yi Yin, Li-Xia Liu, Tian-Bo Ding, Chun-Hong Yang, Guy Smagghe, Yan Shi

**Affiliations:** 1 Key Lab of Integrated Crop Pest Management of Shandong Province, College of Plant Health and Medicine, Qingdao Agricultural University, Qingdao 266109, China; yangliuzhiyi@163.com (Y.L.); 18265024610@163.com (Y.-H.Z.); 17669891160@163.com (C.-H.S.); yinjiayijy@163.com (J.-Y.Y.); tianboding@126.com (T.-B.D.); chyang@qau.edu.cn (C.-H.Y.); 2 Department of Entomology, College of Plant Protection, Northwest A&F University, Yangling 712100, China; 3 Belgorod College of Food Sciences, Dezhou University, Dezhou 253023, China; liulx@dzu.edu.cn; 4 Institute of Entomology, Guizhou University, Guiyang 550025, China; 5 Department of Biology, Vrije Universiteit Brussel (VUB), 1050 Brussels, Belgium

**Keywords:** *Plutella xylostella*, *Phytolacca americana*, fruit extract, botanical repellent, chemical ecology, olfactory perception, electroantennography, integrated pest management

## Abstract

*Phytolacca americana* L. (pokeweed) is an invasive alien plant in China that experiences little herbivore or disease pressure, suggesting the presence of effective chemical defenses. Although insecticidal activity of *P. americana* tissues has been reported, its potential repellent effects against the diamondback moth, *Plutella xylostella* (L.), remain unexplored. In this study, we further optimized distillation conditions to obtain fruit extract (FE) from *P. americana*. Subsequently, its chemical composition was analyzed. behavioral repellency, and antennal olfactory activity against *P. xylostella* adults. Gas chromatography-mass spectrometry (GC-MS) tentatively identified 11 compounds, accounting for 96–99% of the total distillate composition. The major constituents in the final distillate were palmitic acid ethyl ester (26.00%), 6,10,14-trimethyl-2-pentadecanone (25.75%), and ethyl oleate (14.19%). Behavioral assays using a Y-tube olfactometer demonstrated that the FE, as well as diethyl phthalate, palmitic acid ethyl ester, and ethyl oleate, exhibited strong repellent activity against adult *P. xylostella*, whereas 6,10,14-trimethyl-2-pentadecanone showed moderate repellency only at high concentrations (10 μg/μL). Electroantennogram (EAG) recordings revealed robust antennal responses to the three highly repellent compounds, with ethyl oleate eliciting the strongest response (0.58 mV at 100 μg/μL). Furthermore, Diethyl phthalate shows moderate repellency to *P. xylostella*. These results indicate that *P. americana* FE mediates repellency primarily through olfactory perception and that its major constituents contribute substantially to this effect. Overall, this study provides the first laboratory evidence that *P. americana* FE functions as an effective olfactory repellent against *P. xylostella*. The findings offer preliminary proof-of-concept for developing phytogenic repellents.

## Simple Summary

The diamondback moth, *Plutella xylostella*, is a major pest that severely damages cruciferous crops. Controlling this pest has been difficult due to its resistance to many insecticides. In this study, we explored an alternative approach by investigating the repellency of crude extract from the fruit of *Phytolacca americana*, commonly known as pokeweed. We found that the fruit extract has strong repellent effects against the moth, with key compounds like palmitic acid ethyl ester and ethyl oleate playing a major role. Our research shows that the moth avoids these compounds primarily through its sense of smell, making them potential tools for pest management. This discovery offers an alternative to traditional chemical pesticides, which is crucial for sustainable agriculture. These findings may provide a basis for developing natural repellents that help manage *Plutella xylostella* while reducing the environmental impact of pest control strategies.

## 1. Introduction

The diamondback moth, *Plutella xylostella* (L.), is a major pest of Brassicaceae crops worldwide and is among the most extensively studied insect pests due to its global economic impact. Despite decades of research, *P. xylostella* remains one of the most challenging pests to control, owing to its high reproductive capacity, migratory behavior, and remarkable ability to develop resistance to insecticides. Notably, it was the first crop insect reported to develop resistance to microbial *Bacillus thuringiensis* (Bt) insecticides and has since exhibited resistance to nearly every class of chemical insecticides, including the most recent compounds [1,2,3]. In addition to direct feeding damage, *P. xylostella* larvae are vectors for over 30 plant virus diseases, exacerbating their impact on crop yield and quality [4]. Infestation can reduce yield by more than 90%, highlighting the urgent need for effective and sustainable management strategies. Today, chemical insecticides remain the primary tool for controlling *P. xylostella* and other agricultural pests due to their rapid knockdown effect. However, reliance on synthetic chemicals poses multiple challenges, including the development of insecticide resistance, environmental contamination, and reduced safety and quality of agricultural products. Consequently, there is an increasing demand for environmentally friendly and resource-efficient pest management alternatives that align with sustainable agricultural practice [5,6].

Botanical insecticides, particularly plant-derived essential oils, are promising alternatives to synthetic insecticides because they are generally low in toxicity to non-target organisms (NTOs), degrade rapidly in the environment, and reduce chemical residues in food [7]. Plant extracts are complex mixtures of volatile secondary metabolites, including hydrocarbons such as terpenes and sesquiterpenes, and oxygenated compounds such as alcohols, aldehydes, ketones, phenols, and esters [8]. These compounds are typically synthesized in glandular hairs or secretory cavities of leaves, stems, flowers, roots, and fruits, and their composition varies significantly among species, cultivars, and geographic regions [8,9,10]. Many plant extract exhibit strong insecticidal and repellent activity while maintaining low vertebrate toxicity, making them suitable candidates for integrated pest management (IPM) [11,12]. Their effects on insects can include lethality, irritability, repellency, and feeding inhibition, which collectively reduce pest populations and damage [13].

*Phytolacca americana* (L.) or pokeweed is a perennial herb of the Phytolaccaceae family, and native to the Amazon rainforest, but it is now widely distributed globally. Previous studies have demonstrated that extracts from seeds, leaves, and roots of *P. americana* exhibit significant insecticidal activity. For example, saponins from the root system are toxic to common agricultural pests such as red spider mites [14], and berry extracts are lethal to *Oncomelania hupensis*, a freshwater snail of medical importance [15]. These findings suggest that *P. americana* may serve as a valuable source of botanical insecticides with broad-spectrum activity. However, most previous studies have focused on the insecticidal activity against *P. xylostella*, rather than its repellent activity. The fruits of *P. americana* are produced in large quantities, are easily harvested, and can be processed without destroying the entire plant. From an applied perspective, fruit material may therefore offer advantages for potential large-scale development and sustainable utilization. The chemical composition of *P. americana* fruit extract and their behavioral and electrophysiological effects on insect pests, including *P. xylostella*, remain poorly characterized.

The present study aims to address these knowledge gaps by first characterizing the chemical composition of *P. americana* FE using gas chromatography-mass spectrometry (GC/MS). Next, we investigate the behavioral responses of *P. xylostella* to the whole FE and its major components using repellency bioassays. Finally, we employ electroantennography (EAG) to determine the electrophysiological responses of adult moths to these compounds, providing mechanistic insight into olfactory-mediated repellency. We hypothesize that specific volatile compounds in *P. americana* FE elicit strong repellency and/or irritability in *P. xylostella*, offering a basis for developing sustainable pest management strategies. Understanding the chemical ecology of plant-insect interactions has both theoretical and practical significance. From a biological perspective, these results provide new insights into insect olfactory behavior, host preference, and detection of plant secondary metabolites. Practically, identifying plant-derived compounds with high repellency or toxicity toward *P. xylostella* can contribute to the development of environmentally friendly pest management tools. This is particularly relevant in the context of climate change, which is expected to alter pest distribution, crop vulnerability, and the efficacy of traditional insecticides. By exploring botanical alternatives such as *P. americana* FE, the present work supports the broader goal of reducing chemical pesticide use while maintaining crop productivity and environmental sustainability.

## 2. Materials and Methods

### 2.1. Insects

*P. xylostella* adults were obtained from the Shandong Key Laboratory of Integrated Pest Management, College of Plant Health and Medicine, Qingdao Agricultural University, China. All insects were reared in cages (50 cm × 50 cm × 50 cm) at 23 ± 1 °C, 70 ± 10% relative humidity (RH), and a photoperiod of 16 h light followed by 8 h darkness, as described previously [16]. Radish seedlings (7 days old) served as the main food source for larvae, while adults were provided a 10% honey solution daily. Healthy adults were used in all bioassays.

### 2.2. Chemicals

The following synthetic chemicals were purchased from Sigma-Aldrich (St. Louis, MO, USA): diethyl phthalate (≥98%), palmitic acid ethyl ester (≥98%), ethyl oleate (≥99%), and 6,10,14-trimethyl-2-pentadecanone (≥98%).

### 2.3. FE Extraction and Analysis

Fruits of *P. americana* were collected manually in August 2019 from plants located at the foot of a hill in Qingdao, China. Fruits were shade-dried, crushed, and sieved through a 40-mesh screen, then stored at 4 °C. Extraction was completed within 3 months. FEs were obtained via steam distillation using a Clevenger-type apparatus (Glassware Factory of Jiangsu, Yancheng, China), following the previous methods [17], collected low-volatility components, we further optimized the distillation conditions and extended the distillation time to 8 h. Dried, crushed fruit was distilled at 100 °C for 8 h. The resulting fruit extract was dried over anhydrous sodium sulfate and stored in plastic centrifuge tubes at 4 °C. Subsequently, the samples in centrifuge tubes were transferred into brown glass vials with plastic caps for GC-MS analysis. Specifically, using fresh fruits of *P. americana* as raw material, fruit extract was obtained by steam distillation with a yield of 0.32% ± 0.05% (*w*/*w*, n = 3, mean ± standard deviation), calculated on a fresh weight basis.

Quantitative analysis of FEs was performed in quadruplicate using a gas chromatograph (Agilent 6890N series, Santa Clara, CA, USA) equipped with a flame ionization detector (FID). Chromatographic conditions were as follows: fused silica capillary column (30 m × 0.22 mm) with a DB-5 bonded phase (0.25-µm film thickness); carrier gas, N2 at 1.8 mL/min; injector temperature, 220 °C; detector temperature, 240 °C. The column temperature program started at 40 °C (2 min isothermal), increased at 3 °C/min to 240 °C, and was held at 240 °C for 15 min. The injection volume was 1 µL (1% *w*/*v* in CH_2_Cl_2_), with a split ratio of 1:10 and column pressure of 115 kPa.

For qualitative identification, FEs were analyzed using GC-MS (Agilent GCMS-QP 5975B/6890N, Santa Clara, CA, USA). The injector and detector temperatures were 220 °C and 300 °C, respectively. The column temperature was initially held at 40 °C for 3 min and then increased at 3 °C/min to 300 °C, where it was held for 25 min. One microliter of FE (1% *w*/*v* in dichloromethane) was injected in split mode (1:10). Helium was used as the carrier gas at a constant flow rate of 1.8 mL/min. Separation was achieved on an Rtx^®^-5MS capillary column (30 m × 0.25 mm × 0.25 µm, Crossbond^®^ 35% diphenyl-65% dimethyl polysiloxane, Restek Corporation, Bellefonte, PA, USA). Mass spectra were recorded in the range of 29–450 Da using 70 eV ionization energy. Compounds were identified by their relative retention indices and quantified using area normalization.

The sample obtained via steam distillation is defined as fruit extract rather than conventional essential oil, due to differences in raw material treatment, extraction duration and component composition compared with standard essential oil preparation protocols. In addition, it should be noted that no blank control was incorporated in this experiment. We recognize that the absence of blank controls constitutes a major experimental limitation, which makes it difficult to rule out exogenous contaminants and identify potentially other minor peaks.

A total of 11 compounds were putatively identified by GC-MS. Compound matching was performed using the NIST 2017 database (Version 2.2, National Institute of Standards and Technology, Gaithersburg, MD, USA) with a match score threshold of ≥85. Retention indices (RI) were calculated using n-alkane mixtures and further verified with literature-reported RI data. No commercial authentic standards were available for the identified volatile compounds, and key components were comprehensively identified via mass spectral fragmentation characteristics and literature comparison. The relative content of each component was determined by peak area normalization.

### 2.4. Behavioral Repellency Response of FE and Major Compounds

Following GC/MS analysis, three major monomer compounds, namely palmitic acid ethyl ester, ethyl oleate, and 6,10,14-trimethyl-2-pentadecanone, were selected from the *P. americana* FE. Each compound was diluted in hexane at concentrations of 0.01, 0.1, 1, 10, and 100 μg/μL. Hexane alone was used as a negative control. *P. americana* FE was selected for behavioral assays using the Y-tube olfactometer described above. FE was dissolved in acetone at concentrations of 1, 10 and 100 μg/μL.

Behavioral responses of adult *P. xylostella* to the FE and individual compounds were evaluated using a dual-choice Y-tube olfactometer (Glassware Factory of Jiangsu, Yancheng, China). The olfactometer consisted of a Y-shaped glass tube (2.0 cm inner diameter), with each arm and the base 20 cm in length. Each arm was connected to an odor source via a release chamber (2 cm diameter × 20 cm length) (Supplementary Materials). Clean air was split into two streams, each entering a release chamber at 400 mL/min. The Y-tube olfactometer was placed in a fume hood, and a single lamp was positioned directly above the arms to provide uniform illumination.

For each assay, healthy and active adults were individually introduced into the base of the olfactometer and allowed 3 min to make a choice. A choice was recorded when an adult crossed a line 5 cm from the base junction and remained beyond this point for at least 10 s. Adults failing to make a choice (no obvious selection within the specified time or staying in the middle area of the Y-tube) within 3 min were recorded as non-responders. Non-responders were excluded from subsequent statistical analysis. After testing five insects, the Y-tube was replaced with a clean olfactometer, the sides of Y-tube (left/right) were alternated to control for lateral preference (side bias). Then the Y-tube had been washed with 98% ethanol and heated at 70 °C for 20 min. Each treatment included 30 adults, and the assay was repeated 5 times, and a total of 150 adults with each treatment were assayed. Behavioral tests were conducted in a climate-controlled room at 23 ± 1 °C and 70 ± 10% RH between 09:00 and 17:00. The calculation of % repellency has now been clearly defined in the Methods section. Specifically: % Repellency = [(Nc − Nt)/(Nc + Nt)] × 100, where: Nc = number of insects choosing the control arm, and Nt = number of insects choosing the treatment arm.

### 2.5. Antennal Olfactory Responses

Electroantennogram (EAG) recordings were performed using an EAG recording system (Syntech, New Castle, DE, USA) to measure antennal responses of female adults to palmitic acid ethyl ester, ethyl oleate, 6,10,14-trimethyl-2-pentadecanone and diethyl phthalate. Each compound was prepared in hexane at 0.01, 0.1, 1, 10, and 100 μg/μL. Ten microliters of each solution were applied to a 1 cm × 1 cm filter paper disc, which was then inserted into a 15 cm Pasteur pipette, with the tip removed.

Female antennae were excised under a stereomicroscope. The basal segment was trimmed, and the antenna was connected to the ground electrode, with the recording electrode placed at the tip. The odor stimulus pipette was positioned at 1 cm from the antenna. Continuous airflow was maintained at 400 mL/min, and odor pulses of 0.5 s were delivered through the pipette. Hexane alone served as the control. Glass electrodes (Sutter Instrument, Novato, CA, USA) were prepared with 0.1 M KCl and pulled using a horizontal electrode puller. Signals were amplified and recorded using EAG 2000 software (version 2.7, Syntech, New Castle, DE, USA). Six effective replicates were conducted for each compound at each concentration. The relative EAG response was calculated as follows: EAG relative response = 2 × RC∕RC_−1_ + RC_−2_, where RC is the response of the test sample, RC_−1_ is the response of the blank control before testing the sample, and RC_−2_ is the response of the blank control after testing the sample.

### 2.6. Behavioral Repellency Response of Adults Without Antennae

To assess the role of antennal olfaction, three compounds with the strongest repellency at the highest concentration, namely palmitic acid ethyl ester, 6,10,14-trimethyl-2-pentadecanone, and ethyl oleate, were selected. The basal segment of the antenna was excised from adult moths, and behavioral repellency assays were conducted as described in Section 2.4. Thirty adults were used per treatment, and each assay was repeated five times.

### 2.7. Data Analysis

Data were analyzed using SPSS 20.0 (SPSS, Chicago, IL, USA). Behavioral bioassay data from behavioral assays were evaluated using the Chi-square test. EAG responses were analyzed using one-way analysis of variance (ANOVA), with *p* < 0.05 considered statistically significant.

## 3. Results

### 3.1. FE Constituents and Chemical Structures

**Figure 1:**
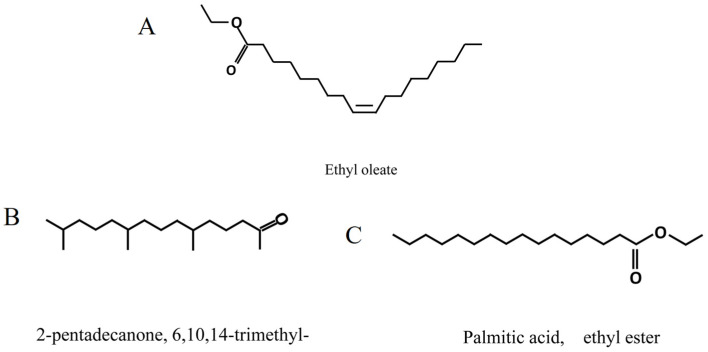
Chemical structures of the three major constituents tentatively identified in the fruit extract (FE) of *Phytolacca americana*. (**A**) Ethyl oleate; (**B**) 6,10,14-trimethyl-2-pentadecanone; (**C**) Palmitic acid ethyl ester.

The yield and chemical composition of *P. americana* fruit FE are summarized in Table 1. The original mass spectra and characteristic ion information of the chromatographic peaks for these chemical components are presented in the Supplementary Materials. A total of 11 compounds were tentatively identified, accounting for 96–99% of the finally distillate composition. Among these, three compounds were selected for further bioassays based on their relative abundance: palmitic acid ethyl ester (26.00%), 6,10,14-trimethyl-2-pentadecanone (25.75%), and ethyl oleate (14.19%). The chemical structures of these three major constituents are shown in Figure 1.

### 3.2. Repellency of P. americana FE Against P. xylostella Adults

**Figure 2:**
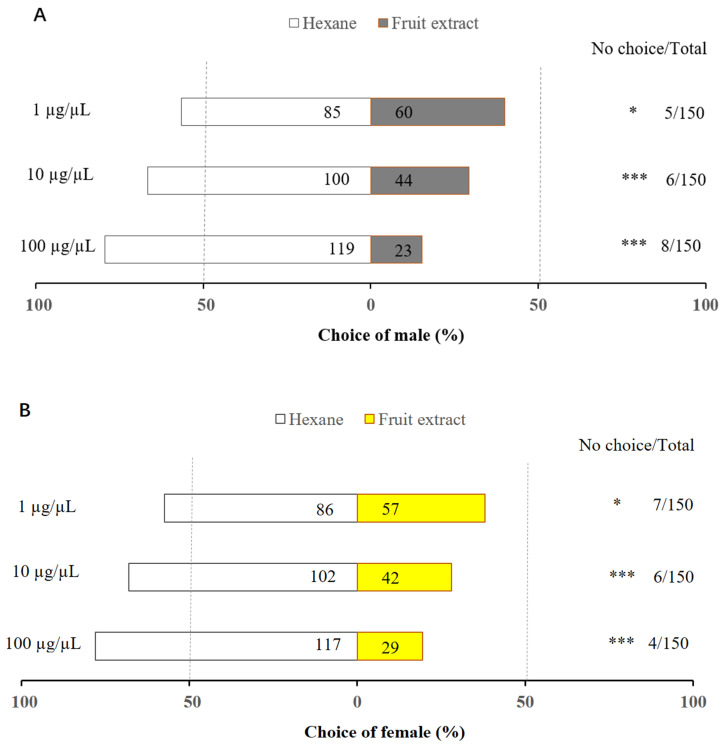
Behavioral responses of *P. americana* FE against *P. xylostella* adults. (**A**,**B**) Y-tube responses of male and female adults to different concentrations of fruit extract. The values in horizontal columns represent the number of choices; Asterisks (*) indicate significant differences (*p* < 0.05); Triple asterisks (***) indicate highly significant differences (*p* < 0.001).

To investigate the repellent effects of final FE distillate against adult *P. xylostella*, we exposed the moths to FE and recorded their choice behavior using a Y-tube olfactometer. The FE from *P. americana* exhibited strong repellent activity against both female and male *P. xylostella* adults (Figure 2A,B), and the repellent activity increased in a concentration-dependent manner. Female adults demonstrated a significant avoidance of fruit extract at 100 μg/μL (χ^2^ = 53.04, df = 1, *p* < 0.001), with 79% choosing Hexane 23% selecting fruit extract. Male adults demonstrated a significant avoidance of fruit extract at 100 μg/μL (χ^2^ = 64.91, df = 1, *p* < 0.001), with 78% choosing Hexane 19% selecting fruit extract. Although a small number of adults showed no preference in the assay, such cases accounted for a very low proportion (2.67–5.33%) of the total and barely affected the experimental results.

### 3.3. Repellency of Major Compounds Against P. xylostella Adults

**Figure 3:**
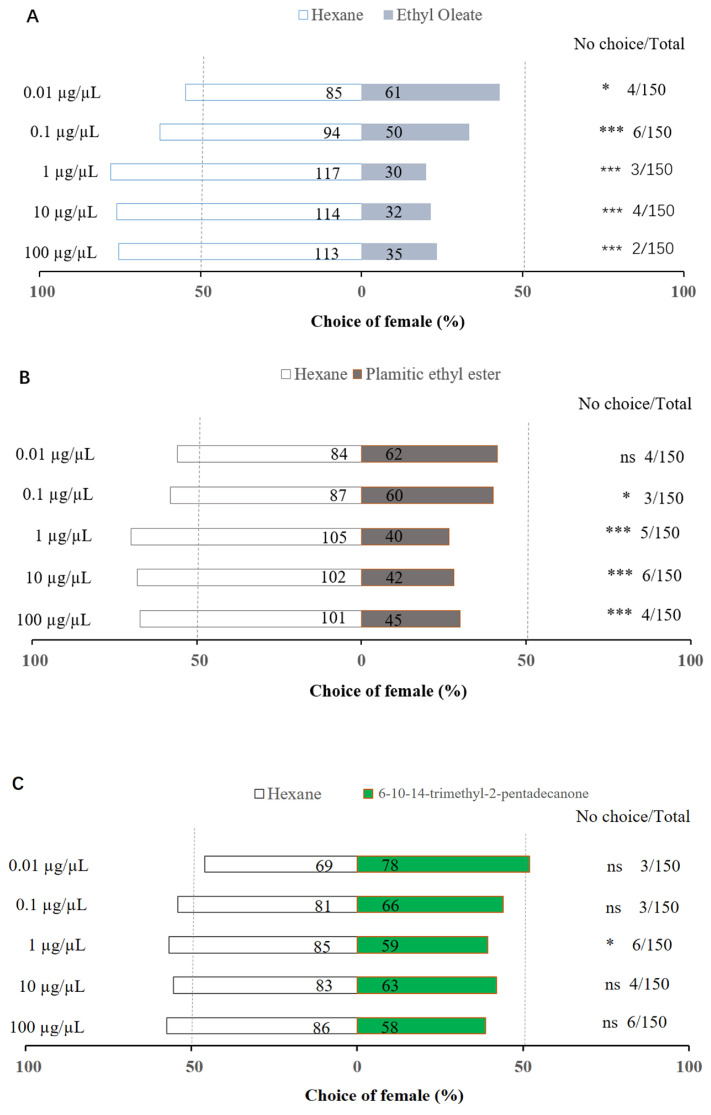
Behavioral responses of three compounds against *P. xylostella* female adults. Y-tube responses of female adults to different concentrations of (**A**) Ethyl oleate, (**B**) Palmitic acid ethyl ester and (**C**) 6,10,14-trimethyl-2-pentadecanone. ns indicates no significant differences (*p* > 0.05); Asterisks (*) indicate significant differences (*p* < 0.05); Triple asterisks (***) indicate highly significant differences (*p* < 0.001).

All three major compounds isolated from final FE distillate xhibited concentration-dependent repellent effects against *P. xylostella* adults (Figure 3A–C).

Ethyl oleate (Figure 3A) showed the strongest repellency among the tested compounds. Female adults demonstrated a significant avoidance of fruit extract at 1 μg/μL (χ^2^ = 41.108, df = 1, *p* < 0.001), with 78% choosing Hexane 21% selecting fruit extract. Palmitic acid ethyl ester (Figure 3B) maintained high repellency across concentrations ranging from 100 to 0.1 µg/µL. Female adults demonstrated a significant avoidance of fruit extract at 1 μg/μL (χ^2^ = 21.48, df = 1, *p* < 0.001), with 70% choosing Hexane 27% selecting fruit extract.

6,10,14-trimethyl-2-pentadecanone (Figure 3C) exhibited the lowest overall repellency among the three compounds. Female adults demonstrated a significant avoidance of fruit extract at 1 μg/μL (χ^2^ = 4.69, df = 1, *p* < 0.05), with 57% choosing Hexane 39% selecting fruit extract.

Overall, the three ester compounds, namely ethyl oleate and palmitic acid ethyl ester, showed stronger repellent activity against *P. xylostella* adults than 6,10,14-trimethyl-2-pentadecanone. The small number of non-responding adults (1.33–4%) had negligible effects on the experimental results.

### 3.4. Antennal Olfactory Responses (EAG)

**Figure 4:**
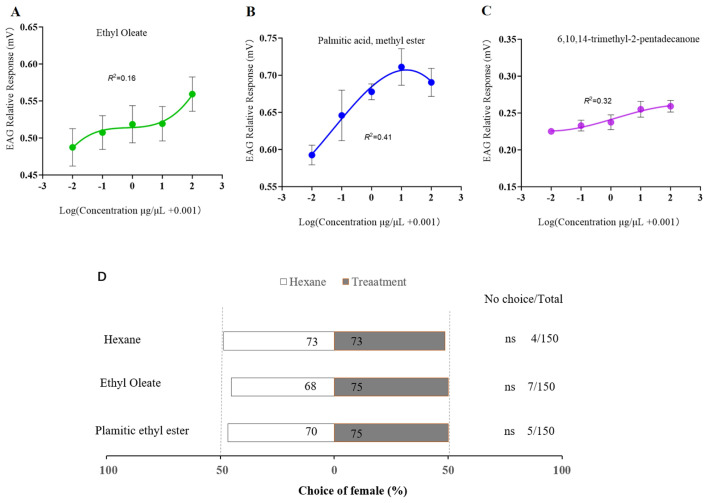
Electroantennogram (EAG) responses and repellency of *P. xylostella* adults to major compounds from *P. americana* FE. (**A**–**C**) EAG responses (mean ± SE) of *P. xylostella* adults to increasing concentrations of (**A**), hexane (**B**), ethyl oleate (**C**), palmitic acid ethyl ester. (**D**), The antennae of *P. xylostella* mediate the selection to ethyl oleate and palmitic acid ethyl ester. ns indicates no significant differences (χ^2^ test, *p* > 0.05), confirming antennae-mediated detection.

EAG responses of *P. xylostella* adults to the three major compounds were recorded across a range of concentrations (Figure 4A–C). Distinct response patterns were observed among the tested compounds.

Ethyl oleate (Figure 4A) showed a positive concentration-dependent increase in EAG response, reaching a plateau at concentrations 100 µg/µL (relative response ≈ 0.56 mV), suggesting stable olfactory perception at higher concentrations.

Palmitic acid ethyl ester (Figure 4B) produced the highest EAG response at an intermediate concentration (10 µg/µL relative response ≈ 0.71 mV), indicating an optimal concentration for antennal stimulation. Responses declined at both lower and higher concentrations.

6,10,14-trimethyl-2-pentadecanone (Figure 4C) elicited the weakest antennal responses among the four compounds. A slight concentration-dependent increase was observed, with a maximum relative response of approximately 0.26 mV at 100 µg/μL.

### 3.5. Repellency Mechanism: Role of Antennae

To assess the contribution of antennal olfaction to repellency, behavioral responses of de-antennated adults to the two most effective compounds, namely ethyl oleate and palmitic acid ethyl ester, were evaluated (Figure 4D).

Removal of antennae resulted in no significant difference in repellency for two compounds compared with hexane treatment. The mean selection rates for de-antennated adults were 46% (χ^2^ = 0.17, *p* > 0.05) for ethyl oleate and 47% (χ^2^ = 0.34, *p* > 0.05) for palmitic acid ethyl ester. These values were close to the theoretical no-repellency threshold (selection rate = 50%).

These results indicate that the repellent effects of the tested compounds are primarily mediated through antennal olfactory perception.

### 3.6. EAG and Repellency Mechanism of Diethyl Phthalate

Diethyl phthalate (DEP) was detected in the final FE distillate of *P. americana*. Subsequent EAG and behavioral assays demonstrated that this compound could elicit antennal responses and exert repellent activity against *P. xylostella*.

Nevertheless, according to existing literature and experimental assessment, DEP rarely occurs as an endogenous constituent in natural plants, with its natural background level generally below the detection limit. Combined with the experimental workflow using various plastic tubes, vials and seals throughout extraction and analysis, we conclude that the detected DEP possibly originated from exogenous plastic contamination, instead of plant biosynthesis.

## 4. Discussion

The diamondback moth, *P. xylostella*, remains one of the most intractable pests of cruciferous crops worldwide due to its extraordinary capacity to rapidly evolve resistance to nearly all major insecticide classes [18,19,20]. This persistent resistance problem has intensified the search for alternative pest management strategies that are effective, environmentally benign, and compatible with resistance management principles. Botanical insecticides, particularly plant FE, are increasingly recognized as promising candidates because of their complex chemistry, rapid environmental degradation, and multi-target modes of action [7,21,22]. In this context, the present study provides novel evidence that *P. americana* FE distillate acts as a strong behavioral repellent against *P. xylostella*, offering both mechanistic insight and applied potential.

Tentative chemical characterization revealed that the final FE distillate is dominated by three major constituents, namely palmitic acid ethyl ester, ethyl oleate, and 6,10,14-trimethyl-2-pentadecanone, together accounting for the majority of the distillate composition. The remaining components accounted for only a minor proportion. Furthermore, compound screening in this study was performed based on previously published reports. This strategy effectively reduced the risk of misidentification caused by compound isomers. Accordingly, we believe that our results are more reliable and convincing. Behavioral assays demonstrated that two of these compounds (palmitic acid ethyl ester, and ethyl oleate) are primarily responsible for the observed repellency. This finding highlights an important innovation of the present work: rather than attributing repellency to the plant extract as a whole, the study identifies specific active constituents and links them directly to behavioral and electrophysiological responses. Such compound-level resolution is essential for developing standardized and reproducible botanical formulations, a major challenge in plant extract-based pest management [9,23]. In addition, it should be noted that the distillation duration was extended to 8 h. Highly volatile components are miscible with lipids and act as carriers to be stripped out together with water vapor during distillation. Meanwhile, long-term hot water immersion of plant matrices causes emulsification and foam entrainment, which carries low-volatility lipids in the form of organic microdroplets and aerosols into the distillate. After pooling all collected fractions throughout the process, low-volatility lipids consequently become the predominant components of the final sample. This study has several limitations. No blank control was included, potential contamination from plasticware was not avoided, and neither standard reference comparison nor spiking verification was performed. Even so, the compound identification results based on mass spectrometry, spectral library matching and literature comparison adequately support the conclusions of this work. Relevant experiments will be added in future studies to improve the overall experimental rigor.

**Figure 5:**
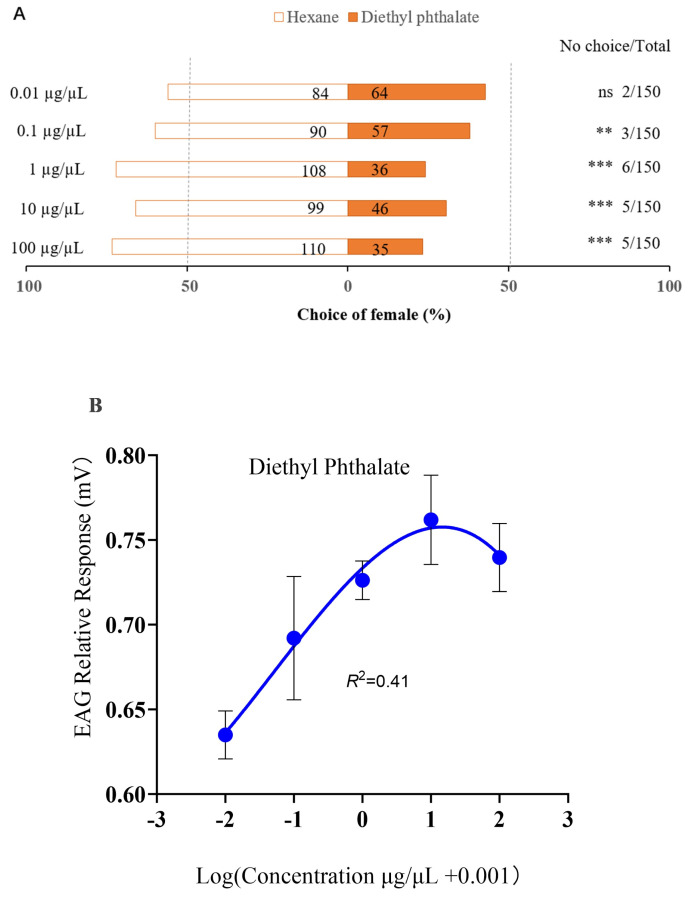
Behavioral responses of diethyl phthalate against *P. xylostella* female adults. (**A**), Behavioral assays of *P. xylostella* adults to diethyl phthalate. ns indicates no significant differences (χ^2^ test, *p* > 0.05); Double asterisks (**) indicate significant differences (*p* < 0.01); Triple asterisks (***) indicate highly significant differences (*p* < 0.001). (**B**), Electrophysiological recordings of *P. xylostella* adults to diethyl phthalate.

Behavioral experiments and EAG experiments have shown that, palmitic acid ethyl ester and ethyl oleate produce significant repellent effect on *P. xylostella* even at low doses. Evidence is accumulating that fatty acid esters are also increasingly recognized as bioactive semiochemicals in plant–insect interactions, influencing host selection, feeding, and oviposition behavior [23,24]. In contrast, the relatively weak repellency of 6,10,14-trimethyl-2-pentadecanone is consistent with previous findings on long-chain ketones, which often exhibit limited volatility and reduced behavioral impact [25]. Together, these results underscore the importance of chemical structure and functional group composition in determining insect behavioral responses. Furthermore, a certain amount of diethyl phthalate (DEP) was also tentative identified in the present experiment (approximately 22% of the total volatile components, the relative content of DEP presented here includes exogenous substances derived from plastic contamination during experimental procedures). Behavioral assays and electrophysiological recordings demonstrated that diethyl phthalate exhibited significant repellent activity against the tested *P. xylostella* (Figure 5). However, we hypothesize that the measurable levels of diethyl phthalate was detected in the samples most likely originated from contamination introduced by plastic consumables during sample preparation and analysis. The reason is that blank controls were not applied in both sample collection and GC-MS procedures. Although trace levels of phthalic acid esters may accumulate in plants via soil absorption, their natural content is typically below 0.01%, which is far lower than the proportion observed in the present study [26]. Such plastic contamination interfered with the analysis of volatile components in the present study to a certain extent.

A key advance of the present study lies in integrating behavioral assays with EAG recordings and antennal ablation experiments to elucidate the sensory basis of repellency. The strong correspondence between behavioral repellency and antennal electrophysiological responses indicates that olfaction is the primary mechanism by which *P. xylostella* perceives and avoids *P. americana* FE constituents. EAG responses to the three major active compounds demonstrate antennal sensitivity to these volatiles, consistent with previous work on plant extract-mediated olfactory detection in lepidopteran pests [23,27]. In addition, the antennal responses of *P. xylostella* to some concentrations of the test compounds were relatively weak in our results, which may be attributed to human interference or experimental variability. These findings contribute to a growing body of research on insect chemical ecology by linking specific plant-derived volatiles to sensory and behavioral outcomes.

Also useful in this study is that the significant reduction in repellency following antennal removal further confirms the central role of antennal olfactory perception. However, repellency was not completely abolished, suggesting that additional chemosensory pathways may contribute. Insects possess gustatory and contact chemoreceptors on appendages such as legs, proboscis, and ovipositor, which can mediate avoidance upon physical contact with deterrent compounds [11,28]. Such multimodal detection systems may enhance the robustness of repellency under variable environmental conditions, a desirable feature for field application [14,29].

At the molecular level, previous studies have identified odorant receptors (ORs) and odorant-binding proteins (OBPs) in *P. xylostella* that mediate avoidance of non-host plant volatiles [30,31,32]. It is plausible that the active plant extract constituents identified here interact with similar ORs, triggering aversive neural signaling pathways [1,33]. Functional validation of these interactions remains a key knowledge gap. Future studies combining heterologous expression, CRISPR-mediated gene editing, and neurophysiological assays would substantially advance understanding of olfactory repellency mechanisms [4,27]. Further studies could further explore the regulatory mechanisms of individual active components in plant crude extracts on insect behavior, growth and development, as well as immune pathways, thereby providing a theoretical basis for the development of green behavioral regulators against insect pests.

From an applied perspective, *P. americana* FE represents a promising tool for sustainable management of *P. xylostella*. Repellents exert lower selection pressure than lethal insecticides, potentially slowing resistance evolution [7,34]. This advantage is particularly relevant under climate change scenarios, where rising temperatures and altered cropping patterns are expected to intensify pest pressure and accelerate resistance development. The identification of key active compounds also facilitates formulation optimization, quality control, and regulatory approval.

Nevertheless, we note here that several limitations should be acknowledged. (1): The absence of standardized blank controls and uncontrolled use of plastic consumables made it difficult to completely exclude exogenous contamination. This issue should be addressed in subsequent experiments by establishing rigorous blank control design and reducing the contact of samples with plastic laboratory supplies. (2): Although the present results were compared with the relevant literature, the occurrence of alternative isomeric forms cannot be definitively excluded. Furthermore, certain compounds may exhibit isomerism. (3): The present study was conducted under controlled laboratory conditions, and FE efficacy may be influenced by environmental factors such as temperature, UV radiation, and rainfall [21,33]. Field trials are therefore essential to validate repellency under realistic agricultural settings. Future work will focus on field and semi-field trials to verify the efficacy under natural field environments. (4): Additionally, plant extract volatility and short persistence may limit long-term effectiveness, highlighting the need for formulation improvements such as microencapsulation or controlled-release systems [17]. (5): Finally, although botanical repellents are generally considered safe, their effects on NTOs, including natural enemies such as *Cotesia plutellae*, must be carefully evaluated to ensure ecological compatibility [35]. These issues were not explored in the current research.

## 5. Conclusions

In conclusion, the present study provides new insights into the chemical ecology of *P. xylostella* by identifying *P. americana* fruit extract as a potent olfactory-mediated repellent and elucidating its key active constituents. Notably, fatty acid ethyl esters and ketones have strong support as plant-derived compounds contributing to repellent effects, whereas phthalate contaminants cannot be completely ruled out. By combining chemical analysis, behavioral assays, and electrophysiological evidence, the work advances both fundamental understanding and practical application of botanical repellents. The laboratory findings provide a preliminary basis for exploring plant-derived repellents and eco-friendly strategies suited to sustainable agriculture and pest resistance management.

## Figures and Tables

**Table 1:** Chemical composition of fruit extract derived from *Phytolacca americana*. Mf, molecular formula; MM, molecular mass; CAS, Chemical Abstracts Service number; Rt, retention time (min); Ri, retention Index; RI_calc_: Calculated retention index; RI_lit_ = Retention Index from literature; Match Score (NIST): Spectrum matching score from NIST database; Identification Method: compound identification based on mass spectrum matching (MS) and retention index (RI) comparison.

Number	Compound	Mf	MM	CAS	Rt (min)	RI_calc_	RI_lit_	Match Score (NIST)	Identification Method	Mean Composition (%)
1	Plamitic acid ethyl ester	C18H36O2	284	628-97-7	27.04	1926	1945	98	MS+RI	26.00%
2	2-Pentadecanone, 6,10,14-trimethyl-	C18H36O	268	502-69-2	26.07	1844	1842	97	MS+RI	25.75%
3	Ethyl Oleate	C20H38O2	310	111-62-6	28.37	2856	2180	98	MS+RI	14.19%
4	Eicosanoic acid	C20H40O2	312	506-30-9	32.79	2557	2380	95	MS+RI	8.10%
5	Hexadecanoic acid, methyl ester	C17H34O2	270	112-39-0	26.91	2258	1915	93	MS+RI	7.93%
6	Ethyl 9-cis,11-trans-octadecadienoate	C20H36O2	308	137142-61-1	29.16	2156	2152	90	MS+RI	7.73%
7	cis-3-Hexenyl tiglate	C11H18O2	182	67883-79-8	17.59	1283	1325	99	MS+RI	4.40%
8	Oleic Acid	C18H34O2	282	112-80-1	26.32	2291	2152	92	MS+RI	3.47%
9	Hexanal	C6H12O	100	66-25-1	5.07	806	779	96	MS+RI	1.24%
10	2-Decanol	C14H22O	158	1120-06-5	13.06	1194	1185	87	MS+RI	0.72%
11	Tetradecanoic acid	C14H28O2	228	544-63-8	20.21	1756	1740	93	MS+RI	0.47%

Note: All compounds were tentatively identified via mass spectrum matching with NIST 2017 database and retention index (RI) calculation; no authentic reference standards were used, and isomer discrimination could not be fully achieved.

## Data Availability

The raw data supporting the conclusions of this article will be made available by the authors on request.

## Supplementary Materials

The following supporting information can be downloaded at: https://www.mdpi.com/article/10.3390/insects17060641/s1.

## Abbreviations

The following abbreviations are used in this manuscript:

*P. americana*	*Phytolacca americana*
*P. xylostella*	*Plutella xylostella*
