# Supplementary material for: Behavioral and Electrophysiological Evidence for Repellency of Phytolacca americana (Pokeweed) Fruit Extract in Plutella xylostella
Source: Insects. 2026 Jun 17;17(6):641. doi: 10.3390/insects17060641 (PMC13301309; doi:10.3390/insects17060641)
Supplement: Supplementary file 1 [file insects-17-00641-s001.zip › insects-4333066-supplementary.pdf]

Diagnostic ion spectra of chemical composition of fruit extract derived from *Phytolacca americana*.

Number 1

Name: Plamitic acid ethyl ester

Formula: $C_{18}H_{36}O_2$

MW: 284 Exact Mass: 284.27153 CAS#: 628-97-7 NIST#: 233204 ID#: 52733 DB: mainlib

Other DBs: Fine, TSCA, EPA, HODOC, NIH, EINECS, IRDB

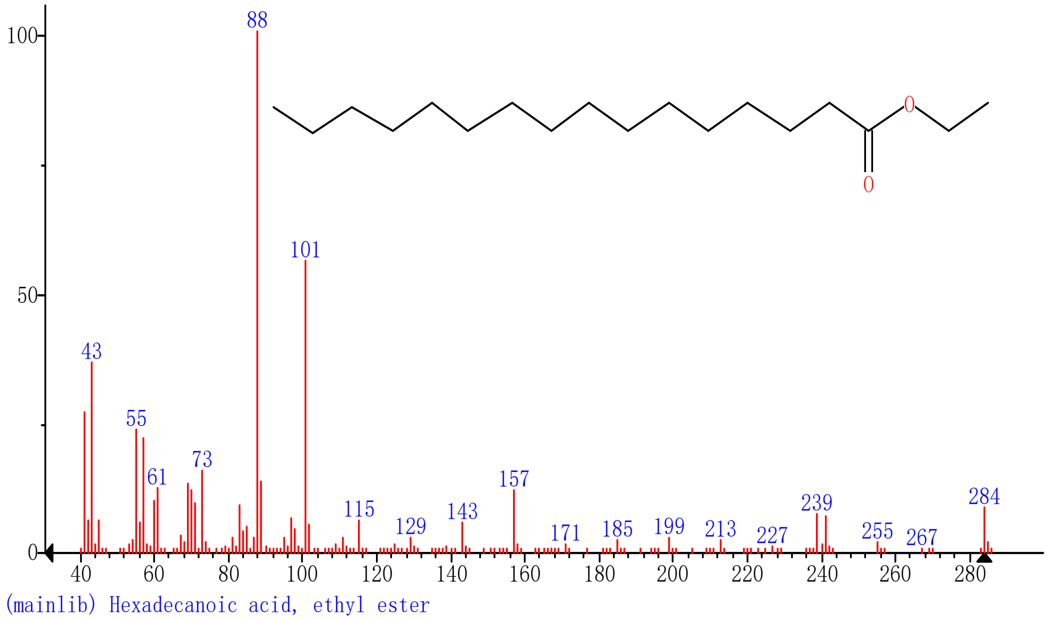

Number 2

Name: 2-Pentadecanone, 6,10,14-trimethyl-

Formula: $C_{18}H_{36}O$

MW: 268 Exact Mass: 268.276615 CAS#: 502-69-2 NIST#: 12976 ID#: 7679 DB: mainlib

Other DBs: TSCA, EINECS

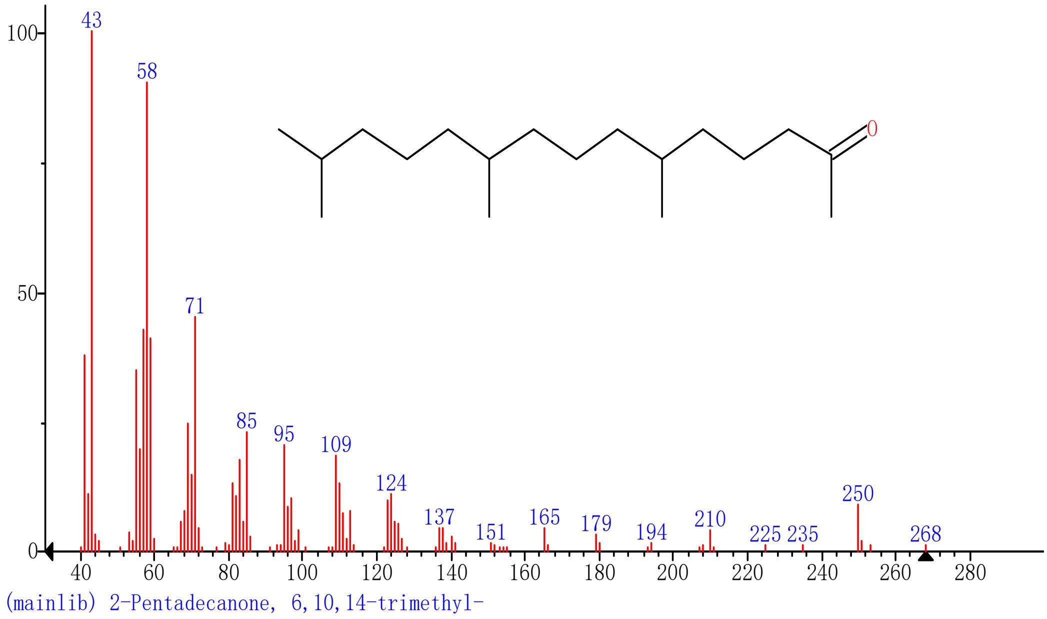

Number 3

Name: Ethyl Oleate

Formula: $C_{20}H_{38}O_2$

MW: 310 Exact Mass: 310.28718 CAS#: 111-62-6 NIST_MSMS

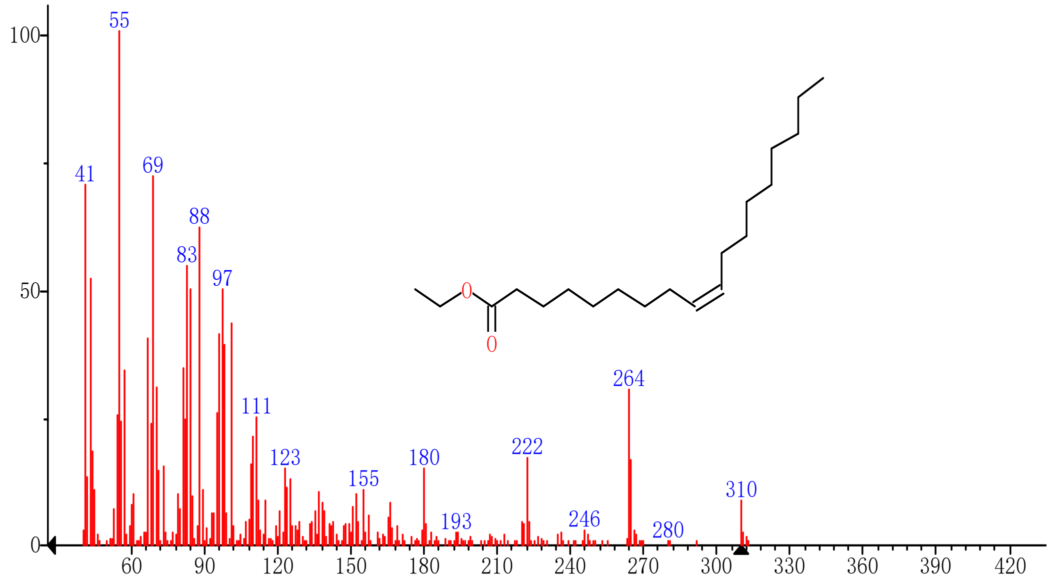

(replib) Ethyl Oleate

Number 4

Name: Eicosanoic acid

Formula: $C_{20}H_{40}O_2$

MW: 312 Exact Mass: 312.30283 CAS#: 506-30-9 NIST_MSMS

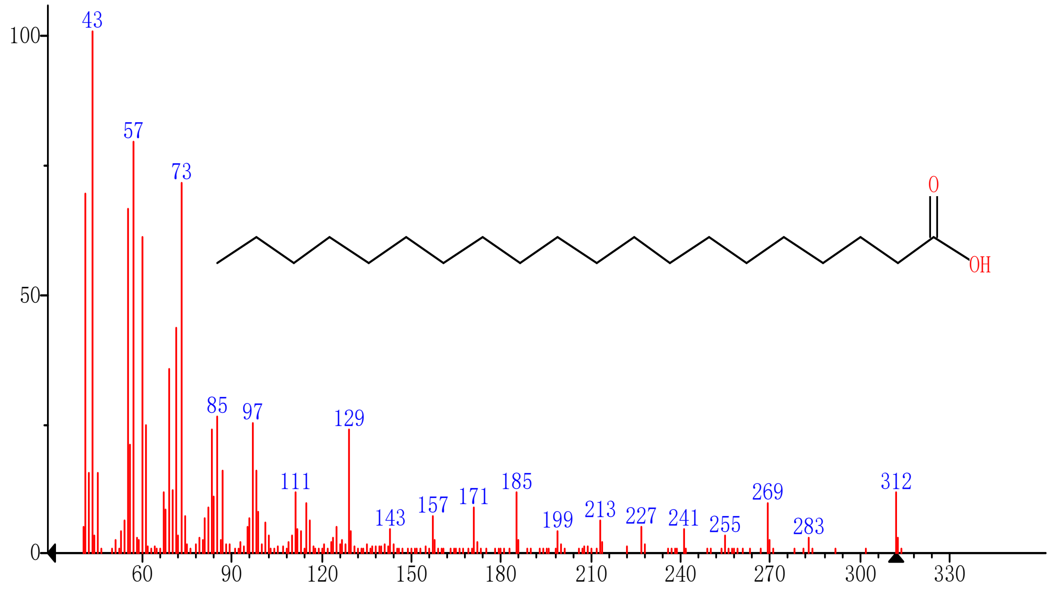

(mainlib) Eicosanoic acid

Number 5

Name: Hexadecanoic acid, methyl ester

Formula: $C_{17}H_{34}O_2$

MW: 270 Exact Mass: 270.25588 CAS#: 112-39-0 NIST#: 42975 ID#: 9767 DB: replib

Other DBs: Fine, TSCA, EPA, HODOC, NIH, EINECS

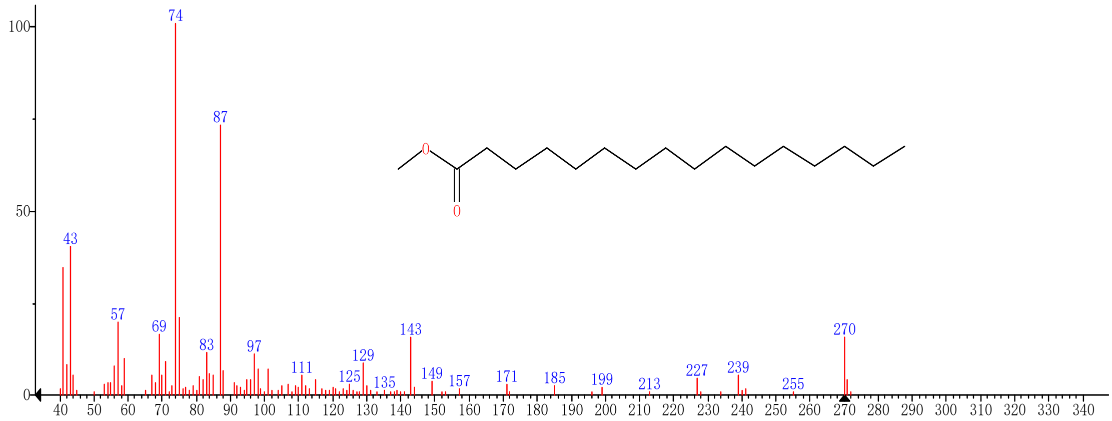

(replib) Hexadecanoic acid, methyl ester

Number 6

Name: Ethyl 9.cis., 11.trans.-octadecadienoate

Formula: $C_{20}H_{36}O_2$

MW: 308 Exact Mass: 308.27153 NIST#: 336698 ID#: 30133 DB: mainlib

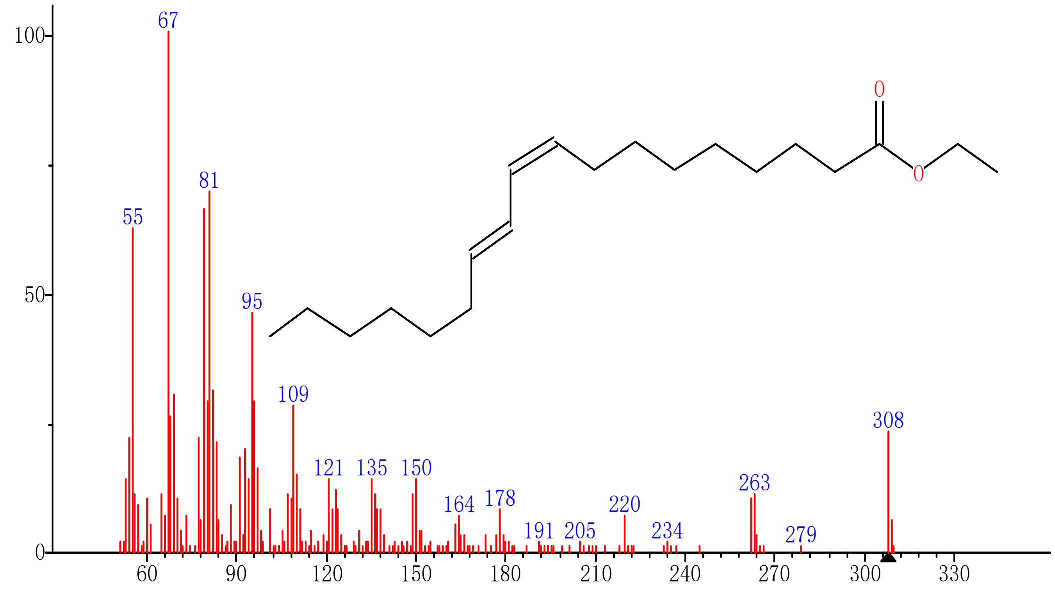

(mainlib) Ethyl 9.cis.,11.trans.-octadecadienoate

Number 7

Name: cis-3-Hexenyl tiglate

Formula: $C_{11}H_{18}O_2$

MW: 182 Exact Mass: 182.13068 CAS#: 67883-79-8 NIST#: 1267315 ID#: 215051 DB: NIST_MSNS

Other DBs: None

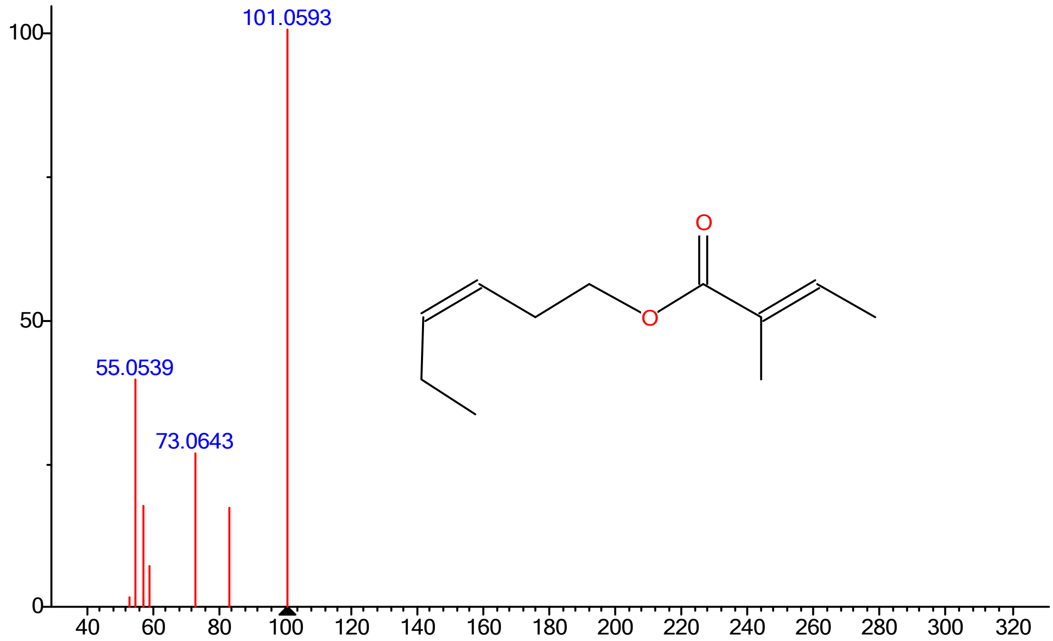

(nist_msms) cis-3-Hexenyl tiglate [M+H-C6H10]+ HCD 8V P=101.1

Number 8

Name: Oleic Acid

Formula: $C_{18}H_{34}O_2$

MW: 282 Exact Mass: 282.25588 CAS#: 112-80-1 NIST#: 154664 ID#: 4486 DB: replib

Other DBs: TSCA, RTECS, USP, HODOC, NIH, EINECS, IRDB

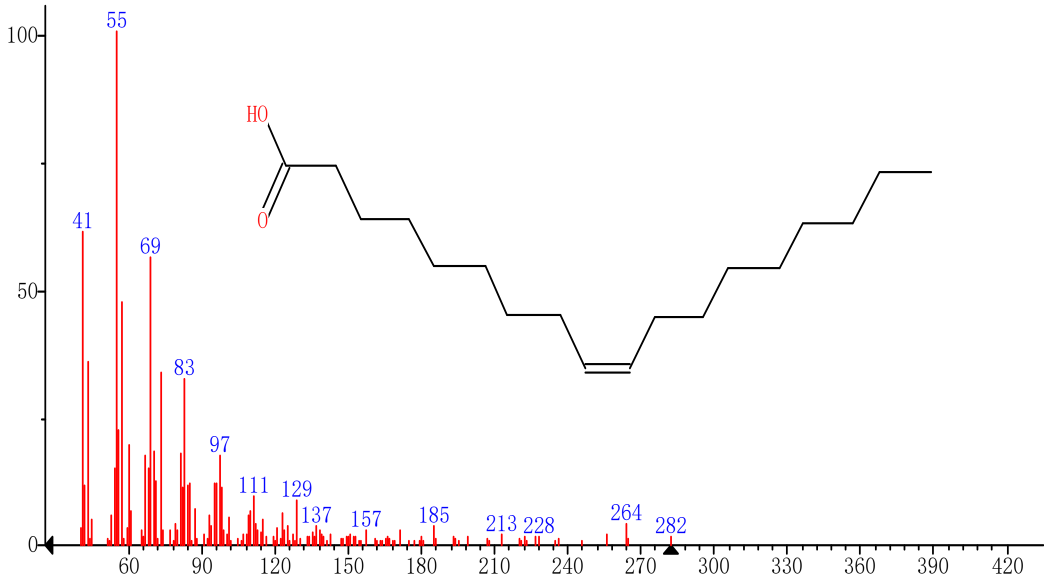

(replib) Oleic Acid

Number 9

Name: Hexanal

Formula: $C_6H_{12}O$

MW: 100 Exact Mass: 100.088815 CAS#: 66-25-1 NIST#: 413762 ID#: 6319 DB: replib

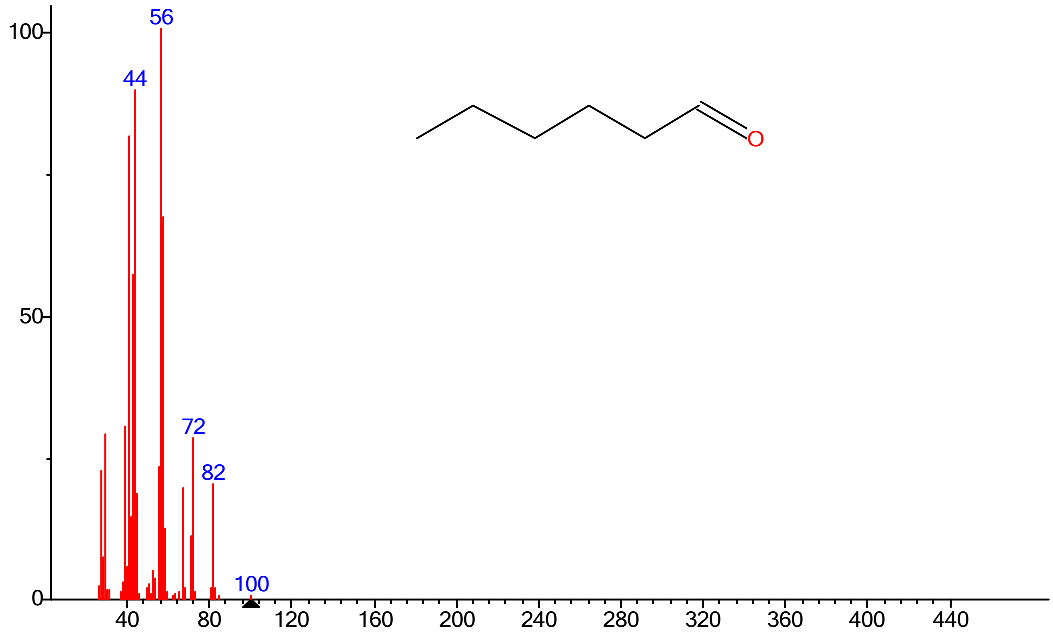

(replib) Hexanal

Number 10

Name: 2-Decanol

Formula: $C_{10}H_{22}O$

MW: 158 Exact Mass: 158.167066 CAS#: 1120-06-5 NIST#: 1071358 ID#: 31091 DB: NIST_MSMS

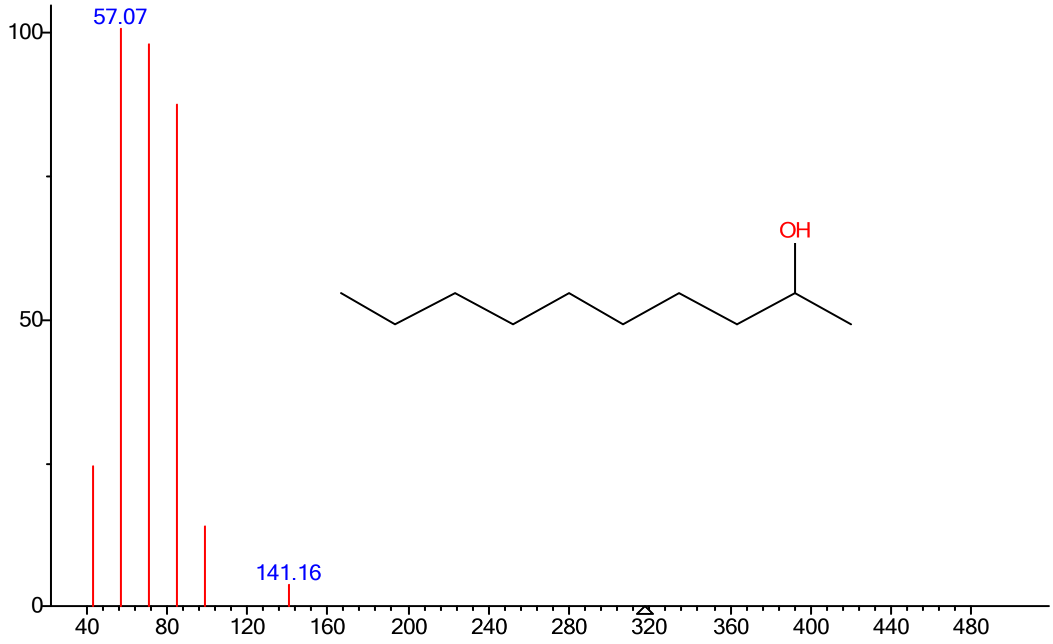

(nist_msms) 2-Decanol [2M+H]+ QTOF 12V P=317.3

Number 11

Name: Tetradecanoic acid

Formula: $C_{14}H_{28}O_2$

MW: 228 Exact Mass: 228.20893 CAS#: 544-63-8 NIST#: 379632 ID#: 11145 DB: replib

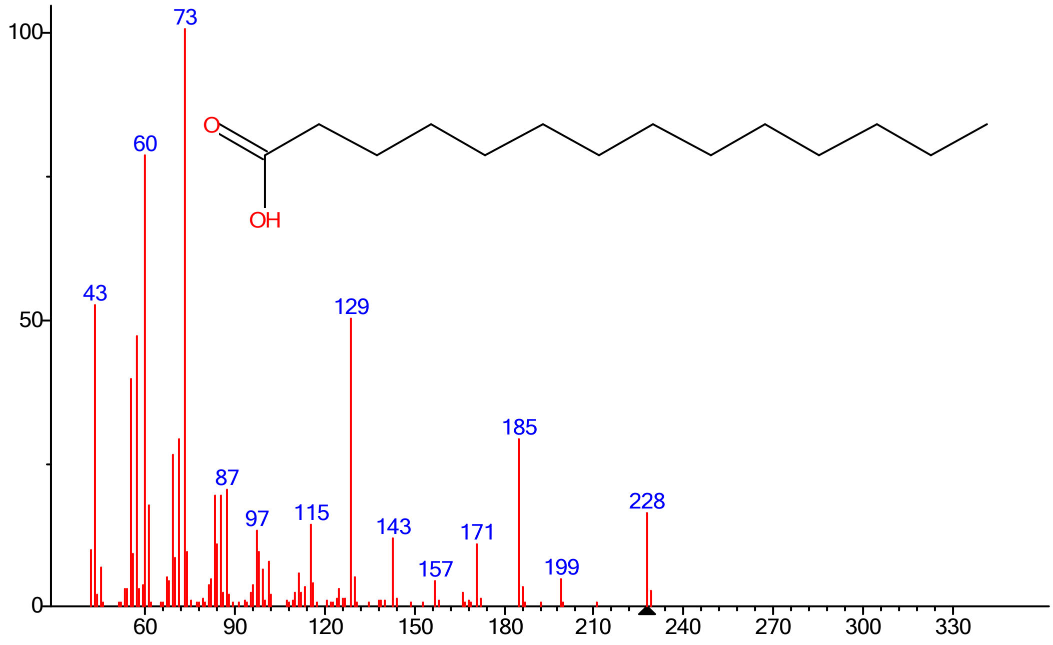

(replib) Tetradecanoic acid

Name: Diethyl Phthalate

Formula: C12H14O4

MW: 222 Exact Mass: 222.089209 CAS#: 84-66-2 NIST_MSMS

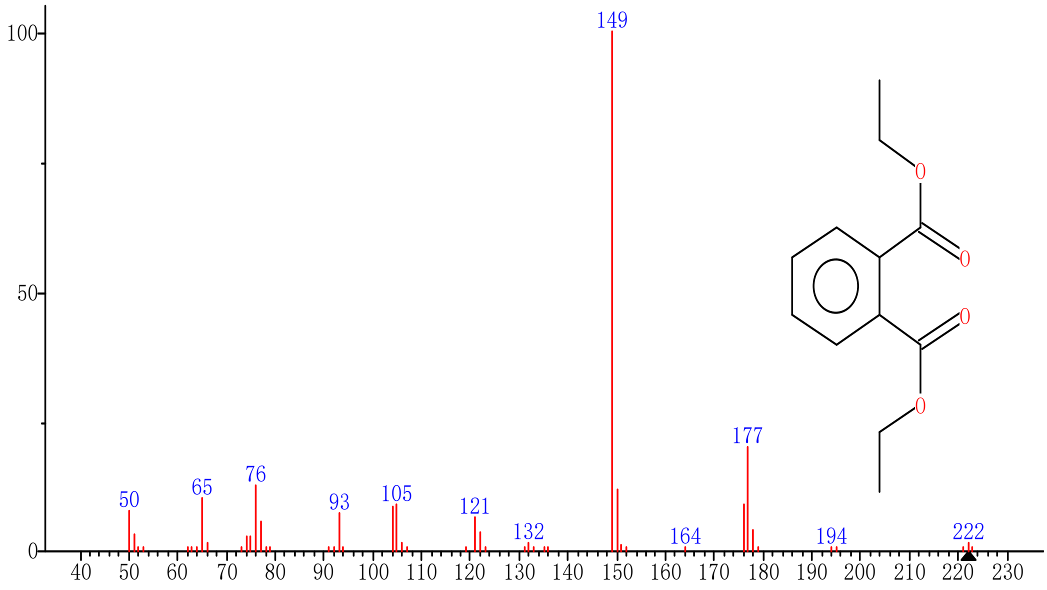

(replib) Diethyl Phthalate
